# Understanding the Dynamic Relationship of Diabetes Distress and Glycemic Indicators in Foot Ulcer Patients: A Correlative Study

**DOI:** 10.7759/cureus.57328

**Published:** 2024-03-31

**Authors:** Anju Ullas, Prabha Adhikari MR, KC Leena, S Sasikumar

**Affiliations:** 1 Department of Medical Surgical Nursing, Yenepoya Nursing College, Yenepoya (Deemed to be University), Mangaluru, IND; 2 Department of Geriatric Medicine, Yenepoya Medical College Hospital, Yenepoya (Deemed to be University), Mangaluru, IND; 3 Department of Community Health Nursing, Yenepoya Nursing College, Yenepoya (Deemed to be University), Mangaluru, IND; 4 Department of Medical Surgical Nursing, Father Muller College of Nursing, Mangaluru, IND

**Keywords:** relationship, patients, foot ulcer, glycemic indicators, diabetes distress

## Abstract

Background: Diabetes-related distress and glycemic indicators are the most common concerns for patients with diabetes mellitus and have a major impact on diabetic patients’ lifestyle, mental well-being, and healthcare access. The principal aim of research in this field is to ascertain the correlation between distress associated with diabetes and glycemic indicators. this helps in developing interventions that can enhance the overall physical and mental well-being of individuals with diabetes.

Objective: The objective is to assess the diabetes distress and glycemic indicators among patients with foot ulcers and to find the correlation between diabetes distress and glycemic parameters.

Materials and methods: A descriptive correlational study was conducted among 159 patients with foot ulcers who were admitted to the hospital by using a non-probability purposive sampling method. The severity of diabetes distress was evaluated utilizing the four-subscale Diabetes Distress Scale (DDS-17). Glycemic indicators are calculated through the assessment of random blood sugar, fasting blood sugar (FBS), and glycosylated hemoglobin (HbA1c).

Result: The study revealed that most participants were above 60 years old and were male. Of the samples, 52% had moderate distress. All the subscales of diabetes distress are correlated to the overall DDS score. A negative correlation (r= -0.162, p < 0.041) was found between emotional burden and FBS which was statistically significant, whereas FBS is positively related to HbA1c (r=0.194, p=0.015).

Conclusion: The significant correlation between DDS scores, the subscales of diabetes distress, and glycemic indicators highlights the criticality of incorporating diabetes distress management into comprehensive strategies for managing diabetes. Moreover, the research underscores the necessity of employing multidisciplinary strategies when attending to diabetic patients to prevent complications.

## Introduction

Diabetes is a long-term metabolic disease that frequently presents with significant complications that affect a person's life [1]. Foot ulcers, a frequent complication, signify a critical issue due to their potential to cause severe morbidity and mortality. Managing these ulcers requires attention not only to their physical aspects but also to the emotional and psychological impact on individuals [2]. An estimated 33 million adults with diabetes worldwide have diabetic foot ulcer (DFU), with a 6.3% global prevalence of the condition according to a recent meta-analysis, in which 80% of these patients, are the primary reason for lower limb amputations [3,4].

Diabetes distress (DD) can have a substantial impact on a patient's compliance with prescribed treatments, lifestyle adjustments, and overall disease management. It is frequently characterized by emotions such as remorse, frustration, or helplessness [5]. Maintaining optimal blood sugar levels isn't just crucial for preventing complications in diabetes; it also significantly influences the healing process of foot ulcers in affected individuals [6].

The importance of glycosylated hemoglobin (HbA1c) in wound healing is controversial, even though diabetes distress has been linked to poor glycemic control [7]. Indeed, certain research indicates a clear correlation between HbA1c levels and the rate at which wounds heal, other studies showed no connection at all between this glycemic control and wound healing [8,9]. The correlation between DD and glycemic indicators, specifically in this subgroup of patients, continues to be a subject that requires thorough investigation.

The study aims to investigate the correlation between DD levels and glycemic indicators among foot ulcer patients, contributing to a deeper comprehension of the factors influencing the management of foot ulcers in individuals with diabetes distress. Such insights could potentially guide healthcare practitioners in devising more holistic approaches to care that encompass both physical and emotional aspects, ultimately improving outcomes for patients managing diabetes-related foot ulcers.

## Materials and methods

Study design

A descriptive correlational study was carried out among diabetes foot ulcer patients admitted to a particular hospital, Mangaluru, to determine the relationship between patients' DD and glycemic indicators.

Study sample

Patients with diabetic foot ulcers were the study's target population. A total of 159 samples were chosen using the non-probability purposive sampling method based on the following inclusion criteria: the patient had to be older than 40 years, grade 2 to 4 of DFU, according to Wagner foot ulcer classification. The Diabetes Distress Scale (DDS-17) was used to measure diabetes distress.

Data collection

The data was collected after obtaining permission from the scientific review board and ethics committee of the institution (YEC-1/2020/034). Informed consent was taken from all the participants. Demographic information like age (in years), gender, educational status, marital status, area of residence, occupation, monthly income (in rupees), family history of diabetes mellitus, years since diagnosed with diabetes mellitus, and hobbies was collected using demographic proforma and clinical information like grade of ulcer, comorbidities and type of comorbidities were collected using clinical proforma.

Diabetes distress was assessed by DDS-17 [10,11] which consists of four subscales: emotional burden, and physician-related distress. regimen-related distress, interpersonal distress. Glycemic indicators like fasting blood sugar, random blood sugar, and HbA1c were measured in a National Accreditation Board for Hospitals & Healthcare Providers (NABH)-accredited laboratory.

Data analysis

Descriptive statistics like frequency and percentage are used to summarize demographic and clinical variables. The Pearson correlation coefficient was used to find the relation between diabetes distress and glycemic indicators.

## Results

The majority of the participants, 84 (52.8%) were above 60 years old and predominantly male, 117 (73.6%). Most of the participants, 73 (45.9%) had primary education. The majority of the participants, 143 (89.9%) were married and hailed from rural areas, 141 (88.7%). In terms of occupation, the majority 58 (36.5%) were self-employed, and the monthly income for most participants 146 (91.8%) was below Rs. 25000. Most of the participants, that is, 70 (44%) were diagnosed with diabetes for three to six years and 85 (53.5%) had a family history of diabetes mellitus. Watching television emerged as the primary hobby among participants, 52 (32.7%) (Table 1).

**Table 1:** Distribution of study groups based on their demographic characteristics The data are expressed in frequency (N) and percentage (%)

		N = 159
Variables		Frequency (%)
Age in years	41 – 50	18 (11.3)
	51 -60	57 (35.8)
	> 60	84 (52.8)
Gender	Male	117 (73.6)
	Female	42 (26.4)
Educational status	No formal education	39 (24.5)
	Primary	73 (45.9)
	Secondary	29 (18.2)
	High school	16 (10.1)
	PUC	2 (1.3)
Marital status	Single	4 (2.5)
	Married	143 (89.9)
	Widow/widower	12 (7.6)
Area of residence	Urban	18 (11.3)
	Rural	141 (88.7)
Occupational status	Private employee	40 (25.2)
	Self-employed	58 (36.5)
	Agriculture	41 (25.8)
	Homemaker	6 (12.5)
Monthly income in rupees	< 25000	146 (91.8)
	25001 – 50000	12 (7.6)
	≥ 50001	1 (0.6)
Years since being diagnosed with diabetes	< 3	47 (29.6)
	3 to 6	70 (44.0)
	> 6	42 (26.4)
Family history of diabetes mellitus	Yes	74 (46.5)
	No	85 (53.5)
Hobbies	Reading	1 (0.6)
	Watching television	52 (32.7)
	Listening to music	24 (15.1)
	Gardening	32 (20.1)
	Cooking	16 (10.1)
	Playing cards	23 (14.5)
	Stitching	6 (3.8)
	Swimming	5 (3.1)

Most of the participants, 84 (52.8%) were categorized as having grade 3 foot ulcers and did not have any comorbidities, 119 (74.8%). Hypertension was the prevailing comorbidity among the participants, 31 (77.5%) with comorbidity (Table 2).

**Table 2:** Distribution of study groups based on their clinical characteristics CKD: Chronic kidney disease, CHF: Congestive heart failure, PVD: Peripheral vascular disease The data are expressed in frequency percentage

		N = 159
Clinical variables		Frequency (%)
Grade of ulcer	Grade 2	75 (47.2)
	Grade 3	84 (52.8)
Comorbidities	Yes	40 (25.2)
	No	119 (74.8)
Types of comorbidities	Arthritis	6 (15.0)
	CKD	2 (5.0)
	CHF	3 (7.5)
	Dyslipidemia	1 (2.5)
	Hypertension	31 (77.5)
	Low back pain	2 (5.0)
	Neuropathy	1 (2.5)
	PVD	3 (7.5)
	Retinopathy	3 (7.5)

DDS was positively correlated (p < 0.05) with subscales of emotional burden, physician-related distress, regimen-related distress, and interpersonal distress (Table 3).

**Table 3:** Relation between the various diabetes distress and subscales of diabetes distress EB: Emotional burden, PRD: Physician-related distress, RRD: Regimen-related distress, ID: Interpersonal distress, DDS: Diabetes distress scale ***very highly significant, p<0.001, test used: Pearson correlation coefficient (r)

Subscale of Diabetes Distress Scale (DDS)		Correlation	
		r	p value
EB	PRD	-0.058	0.471
	RRD	0.050	0.535
	ID	-0.149	0.062
	DDS score	0.576	< 0.001***
PRD	RRD	0.011	0.889
	ID	0.021	0.788
	DDS score	0.445	< 0.001***
RRD	ID	0.049	0.539
	DDS score	0.472	< 0.001***
ID	DDS score	0.339	< 0.001***

**Figure 1:**
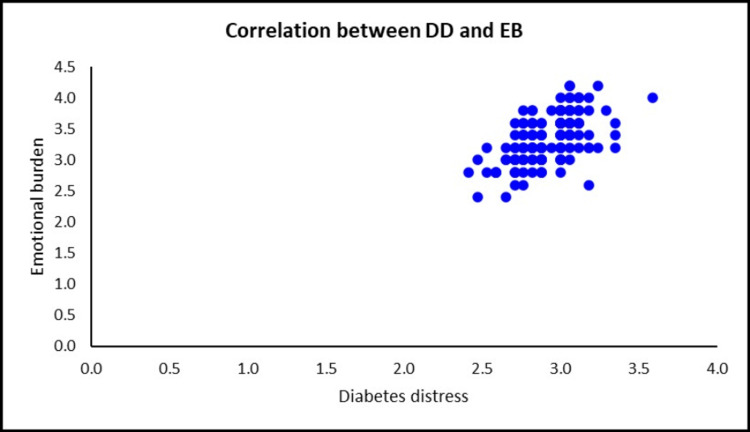
Scatter plot shows the correlation between DD and subscale emotional burden DD: Diabetes distress, EB: Emotional burden

A positive correlation (r= 0.576, p= < 0.001) between diabetes distress and subscale emotional burden (Figure 1).

**Figure 2:**
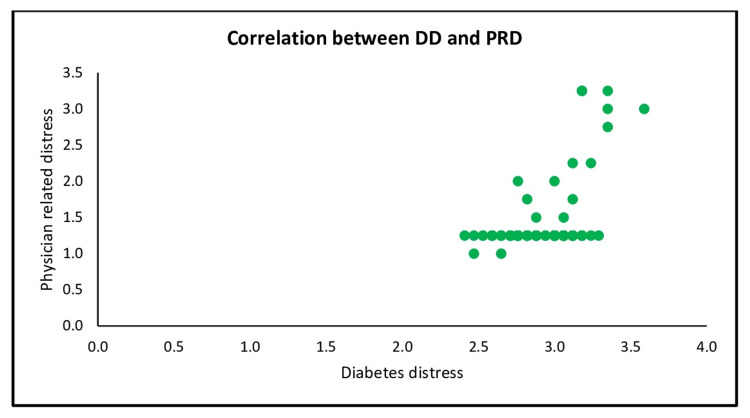
Scatter plot shows the correlation between DD and subscale physician-related distress DD: Diabetes distress, PRD: Physician-related distress

A positive correlation (r= 0.445, p= < 0.001) between diabetes distress and subscale physician-related distress (Figure 2]

**Figure 3:**
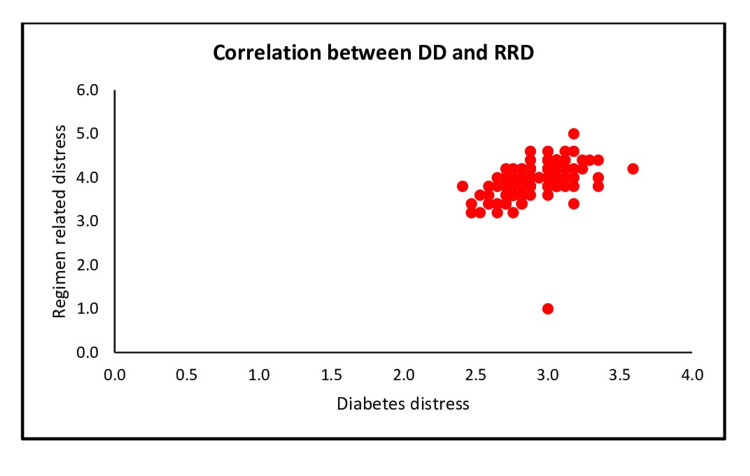
Scatter plot shows the correlation between DD and subscale regimen-related distress DD: Diabetes distress, RRD: Regimen-related distress

A positive correlation (r= 0.472, p= < 0.001) between diabetes distress and subscale regimen-related distress (Figure 3).

**Figure 4:**
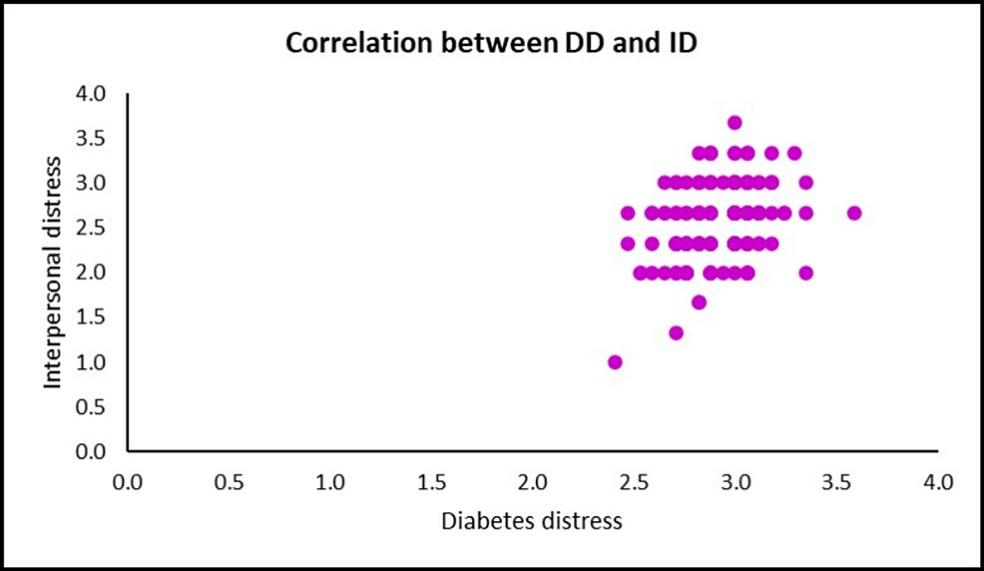
Scatter plot shows the correlation between diabetes distress and subscale interpersonal distress DD: Diabetes distress, ID: Interpersonal distress

A positive correlation (r= 0.339, p= < 0.001) between diabetes distress and subscale interpersonal distress (Figure 4).

A positive correlation (p < 0.05) was found between FBS and HbA1c (Table 4).

**Table 4:** Relation between glycemic indicators FBS: Fasting blood sugar, RBS: Random blood sugar, HbA1c: Glycosylated hemoglobin * Significant p<0.05, test used: Pearson correlation coefficient (r)

Glycemic indicators		r	p value
FBS	RBS	0.018	0.819
	HbA1c	0.194	0.015*
RBS	HbA1c	0.126	0.115

**Figure 5:**
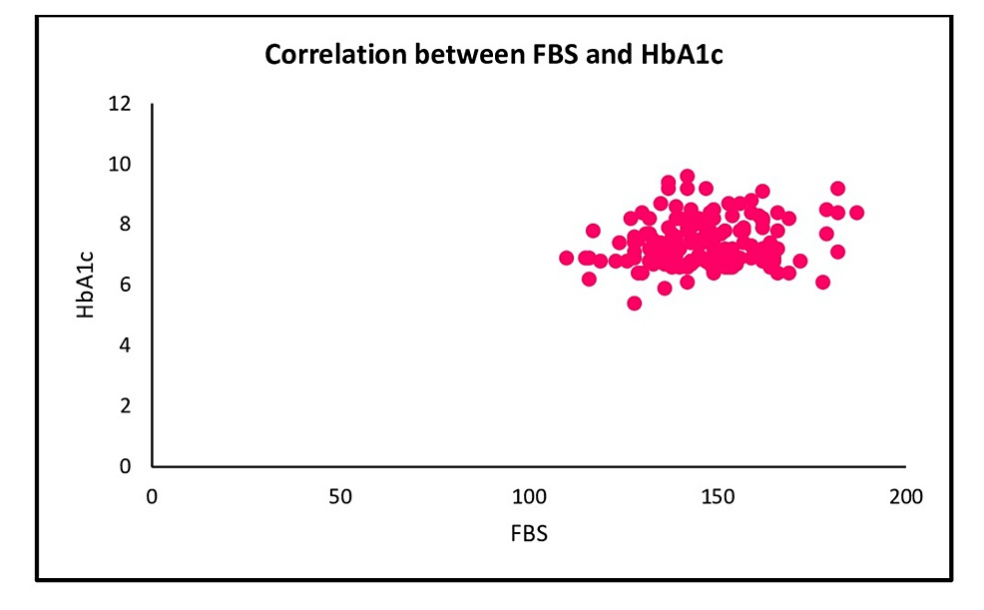
Scatter plot shows the correlation between FBS and HbA1c FBS: Fasting blood sugar, RBS: Random blood sugar, HbA1c: Glycosylated hemoglobin

A positive correlation (r= 0.194, p= 0.015) was found between FBS and HbA1c (Figure 5).

Pearson correlation coefficient (r) was used to find the relation between DDS, FBS, RBS, and HbA1c. There was a negative correlation (p < 0.05) between emotional burden and FBS which was statistically significant (Table 5).

**Table 5:** Correlation between DDS score and glycemic indicators EB: Emotional burden, PRD: Physician-related distress, RRD: Regimen-related distress, ID: Interpersonal distress, DDS: Diabetes distress scale, FBS: Fasting blood sugar, RBS: Random blood sugar, HbA1c: Glycosylated hemoglobin * Significant p<0.05

										N = (159)	
Glycemic indicators	Diabetes Distress Scale (DDS)										
	EB		PRD		RRD			ID		DDS score	
	r	p value	r	p value	r	p value	r		p value	r	p value
FBS	-0.162	0.041*	-0.032	0.689	0.002	0.983	-0.031		0.700	-0.102	0.201
RBS	0.044	0.585	0.008	0.919	0.114	0.152	-0.049		0.539	0.057	0.478
HbA1c	-0.132	0.344	-0.094	0.502	0.170	0.223	-0.168		0.229	-0.081	0.565

**Figure 6:**
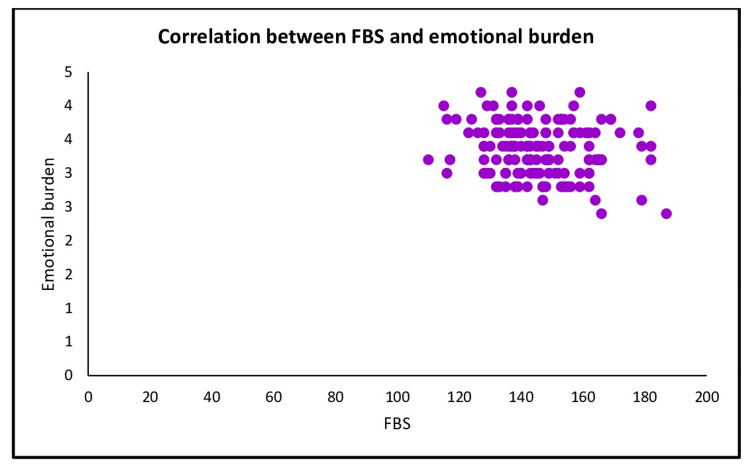
Scatter plot shows the correlation between FBS and diabetes distress subscale emotional burden

A negative correlation (r= -0.162, p= 0.041) between FBS and diabetes distress subscale emotional burden (Figure 6).

## Discussion

This study looked at diabetes distress and glycemic indicators of participants with diabetic foot ulcers. We discovered that HbA1c levels were positively related to fasting blood sugar. This finding also brought to light the connection between fasting blood sugar and emotional burden subscales of diabetes distress.

The current study revealed that 52.2% of patients exhibited moderate diabetes distress which is consistent with another study conducted in Bangladesh (52%), China (57%), and Odisha (42%) [12-14]. DFUs were common among males, present study shows that 73.6% of participants were male, which is supported by another study conducted in Brazil (72.3%) [15].

In the present study, the majority of the participants' diabetes diagnosis duration was three to six years, which is supported by a study that indicates a duration of three to 11 years [12]. A positive correlation (r=0.194, p < 0.05) was found between FBS and HbA1c. It was supported by similar findings in a study conducted in Mysore which shows that FBS values were correlated significantly with HbA1c values (r = 0.6903, p-value <0.0001) [16].

The present study indicates no statistically significant correlation between HbA1c and total DDS score, but similar studies conducted by other researchers showed that there was a statistically significant positive relationship between HbA1c and total DDS score [17]. At the same time, the present study shows that there was a statistically significant negative correlation between FBS and the subscale emotional burden of diabetes distress.

According to recent studies, the health-related quality of life is poorer among patients with DFUs than those without ulcers [18] and the prevalence of amputation is increasing day by day. Patients with more than 20 years of diabetes are more prone to get lower limb amputation [19]. These are occurring because of poor glycemic control.

Diabetes distress and glycemic indicators have a reciprocal relationship that must be duly acknowledged. Glucose regulation can be impacted by diabetes distress; however, elevated distress levels may also be caused by variations in blood glucose levels and the responsibility of diabetes management. To optimize health outcomes and foster holistic well-being among individuals with this chronic condition, it is critical to implement comprehensive care strategies that address both the physiological and psychological dimensions of diabetes. It emphasizes the importance of healthcare professionals taking important measures to prevent diabetes complications.

The limitation of the study was, that the data was collected from a single setting and the population was only patients with DFUs. Diabetes distress may vary due to the duration of the hospital stay.

## Conclusions

The correlation between the distress associated with diabetes and glycemic indicators underscores the importance of incorporating psychological support alongside conventional medical management strategies. By identifying and addressing the psychological repercussions of diabetes, medical professionals can assist patients in overcoming these obstacles and improving their overall health and quality of life.
